# Sepsis and case fatality rates and associations with deprivation, ethnicity, and clinical characteristics: population-based case–control study with linked primary care and hospital data in England

**DOI:** 10.1007/s15010-024-02235-8

**Published:** 2024-04-16

**Authors:** Tjeerd Pieter van Staa, Alexander Pate, Glen P. Martin, Anita Sharma, Paul Dark, Tim Felton, Xiaomin Zhong, Sian Bladon, Neil Cunningham, Ellie L. Gilham, Colin S. Brown, Mariyam Mirfenderesky, Victoria Palin, Diane Ashiru-Oredope

**Affiliations:** 1 Centre for Health Informatics & Health Data Research UK North, Division of Informatics, Imaging and Data Science, School of Health Sciences, Faculty of Biology, Medicine and Health, Vaughan House, The University of Manchester, Manchester Academic Health Science Centre, Manchester, M13 9PL UK; 2 Chadderton South Health Centre, Eaves Lane, Chadderton, Oldham, OL9 8RG UK; 3 Division of Infection, Immunity and Respiratory Medicine, Faculty of Biology, Medicine and Health, The University of Manchester, Manchester Academic Health Science Centre, Manchester, UK; 4 Intensive Care Unit, Manchester University NHS Foundation Trust, Wythenshawe Hospital, Manchester, UK; 5 Healthcare-Associated Infection (HCAI), Fungal, Antimicrobial Resistance (AMR), Antimicrobial Use (AMU) & Sepsis Division, United Kingdom Health Security Agency (UKHSA), London, SW1P 3JR UK; 6 NIHR Health Protection Unit in Healthcare-Associated Infection & Antimicrobial Resistance, Imperial College London, London, UK; 7 Maternal and Fetal Health Research Centre, Division of Developmental Biology and Medicine, The University of Manchester, Manchester, M13 9WL UK; 8 School of Pharmacy, University of Nottingham, Nottingham, NG7 2RD UK

**Keywords:** Sepsis, Primary care, Deprivation, Race, Frailty

## Abstract

**Purpose:**

Sepsis is a life-threatening organ dysfunction caused by dysregulated host response to infection. The purpose of the study was to measure the associations of specific exposures (deprivation, ethnicity, and clinical characteristics) with incident sepsis and case fatality.

**Methods:**

Two research databases in England were used including anonymized patient-level records from primary care linked to hospital admission, death certificate, and small-area deprivation. Sepsis cases aged 65–100 years were matched to up to six controls. Predictors for sepsis (including 60 clinical conditions) were evaluated using logistic and random forest models; case fatality rates were analyzed using logistic models.

**Results:**

108,317 community-acquired sepsis cases were analyzed. Severe frailty was strongly associated with the risk of developing sepsis (crude odds ratio [OR] 14.93; 95% confidence interval [CI] 14.37–15.52). The quintile with most deprived patients showed an increased sepsis risk (crude OR 1.48; 95% CI 1.45–1.51) compared to least deprived quintile. Strong predictors for sepsis included antibiotic exposure in prior 2 months, being house bound, having cancer, learning disability, and diabetes mellitus. Severely frail patients had a case fatality rate of 42.0% compared to 24.0% in non-frail patients (adjusted OR 1.53; 95% CI 1.41–1.65). Sepsis cases with recent prior antibiotic exposure died less frequently compared to non-users (adjusted OR 0.7; 95% CI 0.72–0.76). Case fatality strongly decreased over calendar time.

**Conclusion:**

Given the variety of predictors and their level of associations for developing sepsis, there is a need for prediction models for risk of developing sepsis that can help to target preventative antibiotic therapy.

## Introduction

Sepsis is a life-threatening organ dysfunction caused by dysregulated host responses to infection due to a variety of microorganisms [1]. The dysregulated immune responses lead to an uncontrolled systemic inflammatory response, with resultant tissue and multi-organ dysfunction [2]. Most cases develop sepsis outside the hospital (community-acquired sepsis) while some develop sepsis while hospitalized, often related to invasive devices, procedures, or operations (hospital-acquired sepsis). Although definitions vary between studies, hospital-acquired sepsis cases represent about 10.1% to 53.0% of all sepsis cases [3–8].

The National Health Service (NHS) and UK Health Security Agency (UKHSA) are committed to tackling health inequalities [9]. A recent literature review by our group reported on risk factors for sepsis that were associated with health inequalities [10]. We found that socioeconomic factors associated with increased sepsis incidence included lower socioeconomic status and lower education level. However, findings were not consistent across studies and most of the studies were conducted in the USA. For ethnicity, mixed results were reported [10]. Also, there are only a few studies in literature that have evaluated the incidence and predictors of community-acquired sepsis. The purpose of the study was to measure the association of specific exposures (deprivation, ethnicity, and clinical characteristics) with incident sepsis and case fatality. The approach in this study was data driven without prior hypotheses of specific predictor effects (we refer to predictors as exposure that are associated with the outcome sepsis without implication of causality).

## Materials and methods

### Database

Data sources were the Clinical Practice Research Databank (CPRD) GOLD [11] and CPRD Aurum [12] that contain longitudinal, anonymized, patient-level electronic health records (EHRs) from general practices in the UK. Almost all UK residents are registered with a general practice, which typically provides almost all primary healthcare. If a patient received emergency care (e.g., at Accident and Emergency), inpatient or outpatient hospital care, the general practice of the patient will typically be informed. All UK general practices use EHRs which are provided by several different EHR vendors, including EMIS and Vision. EMIS is the most frequently used primary care EHR [13]. The CPRD GOLD databases includes general practices that use Vision EHR software system, while CPRD Aurum practices use EMIS Web. CPRD GOLD included data on about 11.3 million patients [11] and CPRD Aurum included data on 19 million patients [12]. These databases include the clinical diagnoses, medication prescribed, vaccination history, diagnoses, lifestyle information, clinical referrals, as well as patient’s age, sex, ethnicity, smoking history, and body mass index (BMI). Patient-level data from the general practices were linked to Hospital Episode Statistics (HES), which is a database containing details about hospital admissions. The medical charts with longitudinal information collected during a hospital admission are reviewed and coded using the ICD-10 dictionary by the hospital and clinical codes and dates provided to the HES database. Patient records were also linked to small area deprivation information using socioeconomic information from Index of Multiple Deprivation (IMD) based on the patient’s residential postcode [14]. Patient-level IMD was aggregated into quintiles for the current analysis.

### Study population

This study focused on community-acquired sepsis (most frequent) given the difference in etiology with hospital-acquired sepsis, and on patients aged 65–100 given their higher rates of sepsis. This study was done simultaneously with a study with similar objectives but that used a different English data source [15].

The overall study population consisted of patients aged 65–100 years at any time during the observation period (from January 1, 2000, to July 1, 2020, for CPRD GOLD or up to September 1, 2020, for CPRD Aurum) and who were registered at a GP practice in England. The lower age limit was related to inclusion criteria in the approved protocol; the upper age limit was selected based on the challenges in matching very elderly patients. The practices were restricted to those that contributed to CPRD GOLD or CPRD Aurum and that participated in record linkage. Patient information included sex, age, ethnicity, and medical history. Follow-up of individual patients was defined from the earliest of: (a) their start date of registration with a general practice, (b) prior duration of the patient’s registration in the practice of at least 1 year, or (c) time of reaching age 65 years, until the earliest of: (a) end date due to patients leaving practice, (b) death or (c) time of reaching 101 years of age.

A case–control methodology was selected to measure association between individual exposures and sepsis. Cases were patients who had a hospital record with a sepsis diagnosis (based on the ICD10 codes in HES for sepsis A40 and A41). Only incident cases (i.e., the first sepsis record) were included into study. Each case was randomly matched with up to six controls who had not been hospitalized in the year before. The matching was done by age, sex, calendar time (stepwise by same calendar year and quarter of year, calendar year and then within 5 years), and level of clinical coding in a practice. For each practice, the mean level of coding of clinical information was assessed for each general practice (details are provided elsewhere [16]). Sepsis cases were stratified into community- and hospital-acquired cases. Community-acquired cases were defined as those with a sepsis record within 2 days of the date of hospital admission; hospital-acquired were those that occurred more than 2 days after the hospital admission. Patients were classified at 3-monthly period into four frailty groups (based on the Qfrailty classification). This was based on the Qmortality score [17] (predicting risk of all-cause mortality) in conjunction with the Qadmissions score [18]. Qfrailty was categorized as severe, moderate, minor or non-frailty. The most recent record for frailty prior to the index date was used. Body mass index (BMI), smoking history, and history of 60 clinical conditions prior to the index date were also measured (using code lists from different sources including [19]). Antibiotic exposure in the 2 months before the index date was also measured (as indicator of GP-diagnosed presence of infection).

All-cause mortality outcome for the sepsis cases in the 30 days after the date of sepsis hospital record was assessed using linked death certificates (i.e., case fatality rates). To explore the effects of the age and sex matching on the discrimination between sepsis cases and controls of the logistic models, a second case–control data were also created by only matching cases to controls by calendar time and level of clinical coding in a practice.

The analyses of associations of specific exposures (deprivation, ethnicity, and clinical characteristics) on risk of developing sepsis were conducted in two separate parts. The first one focused on deprivation, ethnicity, frailty, BMI, smoking history, and prior antibiotic exposure. The second one focused on the 60 clinical characteristics. The reason for analyzing the clinical characteristics separately from, e.g., deprivation was that possible causal pathways could be bi-directional (e.g., deprivation could lead to higher incidence of diabetes mellitus but also diabetes could lead to deprivation). With such possible complex causal pathways, adjustment for variables is not preferred statistically.

### Statistical analysis

The matching for age was based on a propensity matching procedure using a caliper (pre-specified maximum difference) of 0.25 of the logit of the propensity score [20]. Greedy nearest neighbor matching was used to select the control unit nearest to each treated unit. Patients were only included once in the analysis. The SAS procedure PSMATCH was used to conduct the matching.

Conditional logistic regression models analyzed the overall effects of individual exposures. Odds ratios (ORs) and 95% confidence intervals (95% CIs) were estimated. Crude ORs assessed the effects of an individual predictor in developing sepsis in matched cases and controls. Adjusted ORs estimated the effects adjusted for other predictors. Random forest (RF) models assessed the relative importance of the 60 clinical characteristics and antibiotic exposure in discriminating between cases and controls; these models predict the probabilities (RF scores) of being a case or control. RF is a supervised tree-based classifier developed by Breiman [21]. Tree-based methods such as RF offer superior performance for sub-group classification over techniques such as logistic regression due to its difficulty to a-priori define the subgroups [22]. A recent study used RF models to identify the medicine combinations associated with higher risks of adverse drug-related hospital admission [23]. The RF models estimated the variable importance index (also known as Gini index) which ranks the explanatory (independent) variables in importance in the tree classifications. We pragmatically selected the maximums of number of trees of 500, depth of 50, and leaf node of 25. Sensitivity analyses were conducted doubling the number of trees and doubling the leaf node. The RF scores were divided into decile groups in the fourth analysis (ranging from low to high risk of developing sepsis) and the distribution of the deprivation, ethnicity, and frailty assessed across these deciles.

Case fatality rates (i.e., 30-days all-cause mortality) were analyzed in unconditional logistic regression models. Crude models evaluated the effects of individual exposures, and adjusted models included all exposures as analyzed for case fatality.

## Results

In the matching process, 99.3% of the sepsis patients were matched to at least one control. Of the matched cases, 94.1% were matched to six controls and 0.2% to one control. 45.1% of the cases were hospitalized during the calendar years 2015–2020. Table 1 shows the characteristics of matched sepsis cases and controls. Cases and controls were well matched on age and sex. The mean age was 80.6 years for cases and 80.4 for controls. For sex, the percentage of women was 50.8% in cases and 51.4% in controls (this small difference in the percentages was related to varying ratios of controls to each case between men and women). Of the 119,529 cases, 108,317 (90.6%) were classified as community-acquired sepsis.

**Table 1:** Characteristics of matched sepsis cases and controls

	Sepsis cases	Controls	Community-acquired sepsis cases	Hospital-acquired sepsis cases
Total *N*	119,529	703,237	108,317	11,212
Age mean (SD)				
Mean	80.6 (8.2)	80.4 (8.1)	80.6 (8.2)	80.7 (8)
Sex *N* (%)				
Women	60,743 (50.8%)	361,564 (51.4%)	55,144 (50.9%)	5599 (49.9%)
Ethnicity *N* (%)				
Black	1585 (1.3%)	9255 (1.3%)	1400 (1.3%)	185 (1.7%)
Mixed	242 (0.2%)	1610 (0.2%)	216 (0.2%)	26 (0.2%)
Other	873 (0.7%)	6388 (0.9%)	800 (0.7%)	73 (0.7%)
South Asian	2375 (2.0%)	13,672 (1.9%)	2152 (2.0%)	223 (2.0%)
Unknown	3816 (3.2%)	65,559 (9.3%)	3474 (3.2%)	342 (3.1%)
White	110,638 (92.6%)	606,753 (86.3%)	100,275 (92.6%)	10,363 (92.4%)
Smoking history *N* (%)				
None	23,080 (19.3%)	132,489 (18.8%)	20,903 (19.3%)	2177 (19.4%)
Current	35,229 (29.5%)	166,651 (23.7%)	31,921 (29.5%)	3308 (29.5%)
Past	16,511 (13.8%)	84,117 (12%)	15,003 (13.9%)	1508 (13.4%)
Unknown	44,709 (37.4%)	319,980 (45.5%)	40,490 (37.4%)	4219 (37.6%)
Frailty *N* (%)				
None	11,406 (9.5%)	139,341 (19.8%)	10,454 (9.7%)	952 (8.5%)
Minor	60,403 (50.5%)	369,212 (52.5%)	54,632 (50.4%)	5771 (51.5%)
Moderate	32,435 (27.1%)	141,920 (20.2%)	29,387 (27.1%)	3048 (27.2%)
Severe	15,285 (12.8%)	52,764 (7.5%)	13,844 (12.8%)	1441 (12.9%)
Deprivation *N* (%)				
Not deprived	25,693 (21.5%)	174,986 (24.9%)	23,356 (21.6%)	2337 (20.8%)
Less deprived	25,489 (21.3%)	162,782 (23.1%)	23,171 (21.4%)	2318 (20.7%)
Moderately deprived	24,045 (20.1%)	144,077 (20.5%)	21,813 (20.1%)	2232 (19.9%)
More deprived	22,798 (19.1%)	121,906 (17.3%)	20,642 (19.1%)	2156 (19.2%)
Most deprived	21,504 (18.0%)	99,486 (14.1%)	19,335 (17.9%)	2169 (19.3%)

As shown in Table 2, severe frailty was strongly associated with the risk of developing community-acquired sepsis (crude OR 14.93; 95% CI 14.37–15.52). The most deprived patients (with deprivation measured by IMD) also showed an increased risk of community-acquired sepsis (crude OR 1.48; 95% CI 1.45–1.51). Non-white races showed lower risks of developing sepsis (crude OR 0.92 in Black people; 95% CI 0.86–0.97). 34.1% of the community-acquired sepsis cases and 11.0% of the controls received an antibiotic in the 2 months before. The presence of infections (as measured by antibiotic exposure in prior two months) was also strongly associated to the risk of community-acquired sepsis (crude OR 4.43; 95% CI 4.36–4.50).

**Table 2:** Crude odds ratios of sepsis by ethnicity, deprivation, frailty, BMI, smoking history, and antibiotic prescribing in prior 2 months stratified by type of sepsis

Characteristic	Sepsis overall	Community-acquired sepsis	Hospital-acquired sepsis
	Crude OR (95% CI)	Crude OR (95% CI)	Crude OR (95% CI)
Deprivation			
Not deprived	Reference	Reference	Reference
Less deprived	1.07 (1.05–1.09)	1.07 (1.05–1.09)	1.06 (0.99–1.12)
Moderately deprived	1.14 (1.12–1.16)	1.14 (1.12–1.16)	1.18 (1.10–1.25)
More deprived	1.29 (1.26–1.31)	1.28 (1.26–1.31)	1.33 (1.25–1.42)
Most deprived	1.49 (1.46–1.52)	1.48 (1.45–1.51)	1.62 (1.52–1.73)
Ethnicity			
White	Reference	Reference	Reference
Black	0.94 (0.89–1.00)	0.92 (0.86–0.97)	1.24 (1.06–1.46)
Mixed	0.83 (0.72–0.95)	0.82 (0.71–0.94)	0.95 (0.62–1.43)
Other	0.75 (0.70–0.81)	0.76 (0.71–0.82)	0.68 (0.53–0.87)
South Asian	1.00 (0.95–1.05)	0.99 (0.94–1.04)	1.13 (0.96–1.32)
Unknown	0.31 (0.30–0.32)	0.31 (0.30–0.33)	0.29 (0.26–0.32)
Frailty			
None	Reference	Reference	Reference
Minor	4.24 (4.13–4.35)	4.20 (4.08–4.31)	4.69 (4.29–5.12)
Moderate	9.94 (9.63–10.26)	9.82 (9.50–10.15)	11.24 (10.11–12.49)
Severe	15.25 (14.70–15.82)	14.93 (14.37–15.52)	18.78 (16.61–21.23)
BMI			
Underweight	1.56 (1.49–1.63)	1.56 (1.49–1.64)	1.53 (1.31–1.78)
Normal	Reference	Reference	Reference
≥ 25–35	0.99 (0.97–1.01)	0.99 (0.97–1.01)	0.94 (0.87–1.00)
≥ 35–40	1.49 (1.43–1.55)	1.5 (1.44–1.56)	1.42 (1.24–1.64)
≥ 40	2.48 (2.34–2.62)	2.49 (2.35–2.64)	2.33 (1.92–2.83)
Missing	0.94 (0.92–0.96)	0.94 (0.92–0.96)	0.92 (0.86–0.98)
Smoking			
Not	Reference	Reference	Reference
Current	1.18 (1.16–1.21)	1.18 (1.15–1.20)	1.23 (1.16–1.32)
Past	1.14 (1.11–1.16)	1.14 (1.11–1.16)	1.14 (1.06–1.22)
Missing	0.76 (0.75–0.78)	0.76 (0.75–0.78)	0.76 (0.71–0.81)
Antibiotic exposure previous 2 months (indicator of presence infection)			
No	Reference	Reference	Reference
Current	4.27 (4.21–4.33)	4.43 (4.36–4.50)	2.92 (2.77–3.06)

Of the 60 clinical characteristics evaluated, strong predictors for community-acquired sepsis included chronic hepatitis (crude OR 2.89; 95% CI 2.72–3.08), being housebound (crude OR 2.66; 95% CI 2.62–2.70), and learning disability (crude OR 3.02; 95% CI 2.68–3.40) (Table 3). Table 4 shows the distribution of frailty, ethnicity, and deprivation by deciles of RF scores (for community-acquired sepsis). The range of predictions by the RF model of being a case ranged from 5.9% in the lowest RF decile to 58.6% in the highest decile. Severe frailty was more prevalent in the highest deciles of the RF score (21.5% in highest decile versus 0% in lowest decile). Deprivation was strongly associated with higher RF probabilities for developing community-acquired sepsis. A logistic model with RF scores as predictors found a c-statistic of 0.788 in the discrimination between sepsis cases and controls.

**Table 3:** Crude odds ratios of developing sepsis for 60 clinical characteristics stratified by sepsis type

	Any sepsis	Community-acquired sepsis	Hospital-acquired sepsis
	Crude OR (95% CI)	Crude OR (95% CI)	Crude OR (95% CI)
Activity limitations	2.29 (2.20–2.38)	2.28 (2.19–2.38)	2.32 (2.04–2.65)
Alcohol problems	2.26 (2.15–2.38)	2.20 (2.09–2.32)	2.86 (2.46–3.33)
Anemia	1.99 (1.96–2.02)	1.98 (1.95–2.01)	2.10 (2.01–2.21)
Anorexia or bulimia	1.54 (1.37–1.72)	1.54 (1.37–1.74)	1.49 (1.05–2.12)
Anxiety	1.28 (1.23–1.34)	1.27 (1.22–1.34)	1.38 (1.19–1.59)
Arthritis	1.29 (1.27–1.31)	1.29 (1.28–1.31)	1.25 (1.20–1.31)
Asthma	1.30 (1.28–1.33)	1.30 (1.28–1.33)	1.30 (1.22–1.39)
Atrial fibrillation	1.82 (1.79–1.85)	1.81 (1.78–1.84)	1.97 (1.86–2.08)
Autoimmune disease	1.64 (1.60–1.67)	1.63 (1.60–1.67)	1.66 (1.56–1.78)
Cancer	1.82 (1.80–1.85)	1.83 (1.80–1.86)	1.76 (1.67–1.84)
Cerebrovascular disease	1.61 (1.58–1.64)	1.61 (1.58–1.64)	1.61 (1.52–1.70)
Chronic kidney disease	2.45 (2.36–2.53)	2.41 (2.32–2.50)	2.78 (2.49–3.11)
Chronic liver + viral hepatitis	2.95 (2.79–3.12)	2.89 (2.72–3.08)	3.46 (2.91–4.12)
Chronic sinusitis	1.02 (0.98–1.06)	1.03 (0.99–1.07)	0.92 (0.81–1.05)
Connective tissue disorder	1.62 (1.58–1.65)	1.61 (1.58–1.65)	1.65 (1.54–1.77)
COPD	1.85 (1.82–1.89)	1.87 (1.83–1.90)	1.75 (1.65–1.86)
Dementia	1.19 (1.17–1.22)	1.22 (1.20–1.25)	0.91 (0.85–0.98)
Depression	1.52 (1.47–1.57)	1.52 (1.47–1.58)	1.52 (1.37–1.70)
Diabetes	1.89 (1.86–1.92)	1.87 (1.85–1.90)	2.04 (1.95–2.15)
Diverticular disease intestine	1.19 (1.16–1.21)	1.18 (1.16–1.20)	1.24 (1.17–1.32)
Dizziness	1.15 (1.13–1.17)	1.15 (1.13–1.17)	1.18 (1.12–1.25)
DVT	1.55 (1.47–1.64)	1.56 (1.47–1.65)	1.51 (1.25–1.82)
Dyspnea	1.92 (1.89–1.95)	1.91 (1.88–1.95)	1.98 (1.88–2.09)
Epilepsy	1.79 (1.72–1.86)	1.80 (1.73–1.87)	1.73 (1.53–1.95)
Falls	1.83 (1.81–1.86)	1.82 (1.80–1.85)	1.92 (1.83–2.02)
Foot problems	1.68 (1.62–1.75)	1.67 (1.60–1.74)	1.84 (1.63–2.08)
Fragility fracture	1.36 (1.33–1.38)	1.35 (1.32–1.38)	1.42 (1.34–1.51)
Hearing impairment	1.10 (1.09–1.12)	1.10 (1.08–1.12)	1.14 (1.08–1.19)
Heart failure	2.25 (2.21–2.29)	2.22 (2.18–2.26)	2.54 (2.39–2.69)
Heart valve disease	1.50 (1.44–1.56)	1.46 (1.40–1.53)	1.84 (1.63–2.08)
Hemiplegia	2.05 (1.94–2.16)	2.05 (1.93–2.17)	2.01 (1.69–2.39)
HIV	0.34 (0.17–0.66)	0.34 (0.17–0.70)	0.29 (0.04–2.20)
Housebound	2.65 (2.62–2.69)	2.66 (2.62–2.70)	2.60 (2.48–2.72)
Hyperlipidemia	1.14 (1.12–1.16)	1.13 (1.11–1.15)	1.20 (1.14–1.27)
Hypertension	1.33 (1.31–1.34)	1.32 (1.30–1.34)	1.39 (1.34–1.45)
Hypotension/syncope	1.47 (1.45–1.50)	1.46 (1.44–1.49)	1.55 (1.46–1.65)
Immunodeficiency disorder	2.48 (2.43–2.54)	2.49 (2.43–2.55)	2.40 (2.22–2.59)
Inflammatory bowel disease	1.57 (1.49–1.64)	1.53 (1.45–1.61)	1.94 (1.66–2.25)
Irritable bowel syndrome	0.91 (0.88–0.94)	0.91 (0.88–0.94)	0.94 (0.85–1.04)
Ischemic heart disease	1.42 (1.40–1.44)	1.41 (1.39–1.43)	1.49 (1.42–1.57)
Learning disability	2.96 (2.65–3.32)	3.02 (2.68–3.40)	2.43 (1.64–3.61)
Memory cognitive problems	1.29 (1.27–1.32)	1.31 (1.28–1.33)	1.19 (1.11–1.27)
Mobility transfer problems	2.08 (2.03–2.13)	2.07 (2.02–2.12)	2.18 (2.01–2.35)
Multiple sclerosis	3.05 (2.79–3.32)	3.12 (2.85–3.42)	2.30 (1.68–3.15)
Osteoporosis	1.31 (1.28–1.34)	1.31 (1.28–1.34)	1.34 (1.25–1.43)
Pancreatitis	1.99 (1.89–2.09)	1.98 (1.88–2.09)	2.04 (1.71–2.43)
Parkinson disease	1.68 (1.62–1.75)	1.69 (1.62–1.76)	1.60 (1.41–1.82)
Peptic ulcer disease	1.38 (1.34–1.41)	1.38 (1.35–1.42)	1.35 (1.24–1.46)
Peripheral vascular disease	1.95 (1.91–1.99)	1.94 (1.89–1.98)	2.07 (1.93–2.22)
Prostate disorders	1.03 (1.01–1.05)	1.03 (1.01–1.06)	0.99 (0.92–1.06)
Psoriasis or eczema	1.20 (1.18–1.22)	1.19 (1.18–1.21)	1.23 (1.17–1.29)
RA connective tissue disorder	1.58 (1.54–1.61)	1.58 (1.54–1.62)	1.57 (1.46–1.69)
Schizophrenia bipolar	1.71 (1.63–1.79)	1.72 (1.64–1.81)	1.64 (1.41–1.92)
Skin ulcer	2.56 (2.51–2.62)	2.55 (2.50–2.61)	2.69 (2.52–2.87)
Sleep disturbance	1.38 (1.35–1.41)	1.38 (1.35–1.41)	1.38 (1.29–1.47)
Social vulnerability	1.32 (1.29–1.36)	1.31 (1.27–1.35)	1.43 (1.32–1.56)
Thyroid disease	1.22 (1.20–1.24)	1.21 (1.19–1.24)	1.28 (1.20–1.36)
Urinary incontinence	1.58 (1.54–1.61)	1.60 (1.56–1.64)	1.36 (1.26–1.47)
Visual impairment	1.29 (1.27–1.31)	1.28 (1.27–1.30)	1.36 (1.30–1.42)
Weight loss anorexia	1.81 (1.73–1.90)	1.80 (1.71–1.89)	1.91 (1.65–2.21)

**Table 4:** Distribution of frailty, ethnicity, and deprivation by deciles of random forest scores for community-acquired sepsis

Characteristic	Decile 1	Decile 2	Decile 3	Decile 4	Decile 5	Decile 6	Decile 7	Decile 8	Decile 9	Decile 10
Predicted probability of being sepsis case mean (SD)	5.9 (1.0)	8.5 (0.9)	11.7 (1.1)	15.8 (1.2)	20.0 (1.2)	24.3 (1.3)	29.4 (1.6)	35.4 (2.0)	44.3 (3.2)	58.6 (5.9)
Frailty: None	45,254 (50.6%)	31,794 (39.1%)	20,334 (23.8%)	12,924 (15.1%)	9223 (10.8%)	6523 (7.6%)	6038 (7.1%)	6233 (7.3%)	5879 (6.9%)	3479 (4.1%)
Minor	39,714 (44.4%)	44,521 (54.7%)	54,494 (63.8%)	54,106 (63.4%)	50,474 (59.1%)	45,172 (52.9%)	41,112 (48.2%)	38,865 (45.5%)	39,105 (45.8%)	35,465 (41.5%)
Moderate	4177 (4.7%)	4651 (5.7%)	9453 (11.1%)	15,745 (18.4%)	20,747 (24.3%)	25,205 (29.5%)	26,363 (30.9%)	26,491 (31%)	26,326 (30.8%)	28,103 (32.9%)
Severe	267 (0.3%)	370 (0.5%)	1094 (1.3%)	2599 (3%)	4932 (5.8%)	8481 (9.9%)	11,853 (13.9%)	13,786 (16.1%)	14,064 (16.5%)	18,328 (21.5%)
Ethnicity Non-white	3725 (5.6%)	3691 (5.2%)	4494 (5.8%)	4064 (5.1%)	3631 (4.5%)	3511 (4.3%)	3473 (4.2%)	3724 (4.5%)	3534 (4.3%)	3433 (4.1%)
White	62,751 (94.4%)	67,770 (94.8%)	72,769 (94.2%)	75,535 (94.9%)	76,427 (95.5%)	77,949 (95.7%)	78,563 (95.8%)	78,807 (95.5%)	79,481 (95.7%)	80,244 (95.9%)
Deprivation: least	24,812 (27.8%)	22,100 (27.2%)	21,633 (25.3%)	21,902 (25.7%)	20,814 (24.4%)	19,776 (23.2%)	19,562 (22.9%)	18,842 (22.1%)	18,452 (21.6%)	17,444 (20.4%)
Less	21,128 (23.6%)	19,464 (23.9%)	20,084 (23.5%)	19,906 (23.3%)	19,480 (22.8%)	19,378 (22.7%)	18,881 (22.1%)	18,719 (21.9%)	18,692 (21.9%)	17,978 (21.1%)
Moderately	18,177 (20.3%)	16,836 (20.7%)	17,548 (20.6%)	17,559 (20.6%)	17,437 (20.4%)	17,429 (20.4%)	17,376 (20.4%)	17,387 (20.4%)	17,367 (20.3%)	17,226 (20.2%)
More	14,427 (16.1%)	13,275 (16.3%)	14,729 (17.3%)	14,493 (17%)	15,076 (17.7%)	15,487 (18.1%)	15,830 (18.5%)	15,948 (18.7%)	15,944 (18.7%)	16,523 (19.4%)
Most	10,868 (12.2%)	9661 (11.9%)	11,381 (13.3%)	11,514 (13.5%)	12,569 (14.7%)	13,311 (15.6%)	13,717 (16.1%)	14,479 (17%)	14,919 (17.5%)	16,204 (19.0%)

All-cause mortality within 30 days was found to be high in community-acquired sepsis cases (Table 5). Severely frail patients had a case fatality rate of 42.0% while non-frail patients had a rate of 24.0% (crude OR 2.30; 95% CI 2.17–2.43, adjusted OR 1.53; 95% CI 1.41–1.65). Sepsis cases with antibiotic exposure in the prior 2 months were less likely to die compared to sepsis not using antibiotics (crude OR 0.71; 95% CI 0.70–0.73, adjusted OR 0.74; 95% CI 0.72–0.76). Case fatality rates strongly decreased over calendar time. The adjusted OR for a yearly change in sepsis mortality was 0.94 (95% CI 0.94–0.95).

**Table 5:** Crude odds ratios of all-cause mortality within 30 days after hospital admission for community-acquired sepsis for age, sex, calendar time, deprivation, ethnicity, frailty, and antibiotic exposure in prior 2 months

	Percentage of sepsis cases dying within 30 days	Crude OR (95% CI)
Age		
60–69	22.7%	Reference
70–79	28.7%	1.37 (1.31–1.44)
80–100	39.4%	2.21 (2.12–2.31)
Sex		
Men	31.9%	Reference
Women	36.0%	1.20 (1.17–1.23)
Calendar time		
2000–2004	44.0%	Reference
2005–2009	42.8%	0.95 (0.91–1.00)
2010–2014	35.4%	0.70 (0.67–0.73)
2015–2020	27.2%	0.48 (0.46–0.50)
Deprivation		
Least	32.3%	Reference
Less	34.2%	1.09 (1.05–1.13)
Moderate	34.0%	1.08 (1.04–1.12)
More	34.7%	1.11 (1.07–1.16)
Most	34.9%	1.12 (1.08–1.17)
Ethnicity		
White	33.4%	Reference
Black	26.2%	0.68 (0.60–0.76)
Mixed	25.3%	0.75 (0.56–1.01)
Other	32.3%	0.95 (0.82–1.10)
South Asian	27.3%	0.71 (0.64–0.79)
Frailty		
None	24.0%	Reference
Minor	31.8%	1.48 (1.41–1.55)
Moderate	37.7%	1.92 (1.83–2.02)
Severe	42.0%	2.30 (2.17–2.43)
Antibiotic exposure in previous 2 months		
No	36.5%	Reference
Yes	29.2%	0.71 (0.70–0.73)

## Discussion

This study found that severe frailty was strongly associated with the risk of developing sepsis. The most deprived patients also showed a 48% increased sepsis risk. Other strong predictors for developing sepsis included antibiotic exposure in prior 2 months, being house bound, having cancer, a skin ulcer, or diabetes mellitus. Fatality rates of sepsis were high and much higher in severely frail patients compared to non-frail patients. Sepsis cases with recent prior antibiotic exposure were less likely to die compared to non-users. Case fatality strongly decreased over calendar time.

There are several limitations in this study. The first was that the sepsis diagnosis was based on coded data (as done by each hospital at discharge or death within the hospital without clinical details of severity or the specific criteria supporting the evidence of the sepsis diagnosis). The diagnosis criteria for sepsis have also changed over the last 2 decades and this study could not apply the latest criteria for sepsis diagnosis. However, sensitivity analyses showed only small effects of the ORs of sepsis with ethnicity, deprivation, and frailty. Also, coding quality may vary between hospitals [24], although it is likely that any misclassification may be random and non-differential leading to underestimates of associations. Another limitation was that this study used broad categories for ethnicity and deprivation, while these characteristics involve heterogenous patient groups with diverse drivers for the incidence of sepsis. The study was observational, and patients could not be randomized between different categories, so we could not separate between direct causal effects of, e.g., ethnicity and indirect effects through higher prevalence of causal factors in these groups. This study assessed the calibration of logistic models. As this analysis was based on a case–control study, the results cannot be generalized to performance in the general population as the rate of the outcome sepsis is very different in a population compared to a case–control setting.

Most published studies on sepsis were hospital-based with limited data on prior medical history and without population-based controls. No studies on community-acquired sepsis were conducted in the UK with the exception of our recent study that used OpenSAFELY and included all ages and covered recent calendar time [15]. In this study of about 250,000 sepsis cases (about 80% were community-acquired), similar results were found. Socioeconomic deprivation and comorbidity were associated with an increased odds of developing non-COVID-19-related sepsis and 30-day mortality in England [15]. With respect to deprivation, four population-based studies on sepsis incidence were found in the literature (other than our recent OpenSAFELY study). All reported increased rates of sepsis incidence with deprivation [25–28]. Two of these studies did not differentiate between hospital- and community-acquired sepsis, which often have different causes and predictors. The two other studies did evaluate community-acquired sepsis, although they included only about 3500 sepsis cases [27, 28]. A prospective cohort with 30,000 US participants also reported a risk prediction model for the development of community-acquired sepsis. It included a smaller number of clinical risk factors such as chronic lung disease, peripheral artery disease, diabetes, stroke, atrial fibrillation, coronary artery disease, hypertension, and deep vein thrombosis [29]. The strength of the present study is that it included a large number of clinical risk factors for a large number of sepsis cases. There is an urgent need to improve our understanding of risk factors for community-acquired sepsis (which in this study involved about 90% of all sepsis cases). As outlined by Kempker et al. sepsis could be viewed as a preventable challenge that can be addressed with population and system-based solutions, including management of risk, factors, appropriate and risk-proportionate antibiotic usage, public awareness, hygiene, and immunization [30].

The National Institute for Health and Care Excellence (NICE) in England has developed a guideline for the recognition, diagnosis, and early management of sepsis [31]. It individually lists patient groups at higher risk of developing sepsis. Most of these are chronic risk factors (such as elderly age, impaired immunity) or include those that affect a substantive number of patients (such as diabetes or other comorbidities). The challenge is that the pathogenesis of sepsis is rapid, and interventions need to be targeted to early triggers of deterioration. A recent review looked at studies of sepsis triggers and tools to support better recognition in healthcare settings. Only 17.7% of identified studies concerned pre-hospital settings [32] and most of those concerned screening by paramedics [33]. Furthermore, some existing tools, such as the Modified Early Warning System (MEWS) [34], Robson criteria [35], Simple Sepsis Early Prognostic Score [36], and a machine learning model [37], mostly concern physiological measurements to support earlier recognition of acute decline. Another widely used tool is NEWS-2 which uses routinely recorded physiological measurements, already recorded in routine practice [38]. However, this tool has not been validated in primary care settings [39]. While these tools focus on early recognition of sepsis in hospital setting [40], there is a lack of monitoring tools that have been tested and can be used at home by patients at high risk of developing sepsis to facilitate earlier contact with the healthcare system. Remote patient monitoring has been used in patients with COVID-19 for early identification of deterioration [41].

The implication of this study is that there is a need for prediction models for risk of developing sepsis that can help to target preventative antibiotic therapy. Important predictors included frailty, deprivation, people with learning difficulties and conditions such as diabetes mellitus and being house bound. The finding of frailty being a major predictor for development of sepsis suggests that interactions between different conditions likely impact the risk of sepsis. The most important predictor in our risk stratification, as expected, was an indicator of infection (antibiotic use in prior two months). Thus, there is a need for developing risk prediction models that consider not only chronic diseases but also, importantly, the acute early triggers and details on infection severity.

In conclusion, the development of community-acquired sepsis is strongly associated with socioeconomic deprivation and some clinical characteristics. Strong predictors of sepsis included recent prior antibiotic exposure, frailty, and conditions such as diabetes mellitus and being house bound. Case fatality rates of community-acquired sepsis were high, particularly in severely frail patients. Given the variety of predictors and their level of associations for developing sepsis, there is a need for prediction models for risk of developing sepsis that can help to target preventative antibiotic therapy.

## Data Availability

Electronic health records are, by definition, considered ‘sensitive’ data in the UK by the Data Protection Act 2018, and cannot be shared via public deposition because of information governance restrictions in place to protect patient confidentiality. Access to data is available only once approval has been obtained through the individual constituent entities controlling access to the data. The data can be requested via application to the Clinical Practice Research Datalink (www.cprd.com).
